# Global Trial Representation and Availability of Tyrosine Kinase Inhibitors for Treatment of Chronic Myeloid Leukemia

**DOI:** 10.3390/cancers16162838

**Published:** 2024-08-14

**Authors:** Mycal Casey, Lorriane Odhiambo, Nidhi Aggarwal, Mahran Shoukier, K. M. Islam, Jorge Cortes

**Affiliations:** 1 Division of Hematology-Oncology, MedStar Georgetown University Hospital, Washington, DC 20007, USA; mycal.casey@medstar.net; 2 Department of Biostatistics, Data Science and Epidemiology, Augusta University, Augusta, GA 30912, USA; 3 Department of Medicine, Medstar Georgetown University Hospital, Washington, DC 20007, USA; 4 Georgia Cancer Center, Augusta University, Augusta, GA 30912, USA; 5 Medical College of Georgia, Augusta University, Augusta, GA 30912, USA

**Keywords:** chronic myeloid leukemia, global health, tyrosine kinase inhibitors

## Abstract

**Simple Summary:**

We aimed to evaluate the extent to which clinical trials represent countries with different socio-demographic indexes and the availability of tyrosine kinase inhibitors for chronic myeloid leukemia. We found a significant lack of availability for tyrosine kinase inhibitors on the global level, and a lack of equitable distribution of trials across countries is an issue that needs to be addressed.

**Abstract:**

**Background**: Evaluating clinical trial representation for countries with different socio-demographic index (SDI) and tyrosine kinase inhibitor (TKI) availability for chronic myeloid leukemia (CML). **Methods**: CML incidence rates (IRs) and disability-adjusted life years (DALYs) (1999–2019) from the Institute of Health Metrics and Evaluation were analyzed. Trials investigating TKI use in CML were obtained from ClinicalTrials.gov. Site data for eligible trials (N = 30) and DALYs were analyzed. TKI approvals, DALYs, and IRs were summarized by SDI. **Results**: North America (NA) had significant decreases in annual percent change (APC) in DALYs and incidence rates from 1999 to 2004. IRs were highest in Europe and Central Asia (ECA) and NA, while DALYs were highest in South Asia (SAsia) and Sub-Saharan Africa (SSA). Countries in the high-SDI quintile were likely to have lower DALYs than lower-SDI quintiles. Differences in regional DALYs vs. sites in TKI trials were significant for SAsia, SSA, and ECA. High-SDI countries were included in all 30 trials, and TKI approvals were prominent in high-SDI (142) vs. low-SDI (14) countries. **Conclusions:** The inclusion of disproportionately affected countries during the design of and recruitment into clinical trials should occur, as should TKI availability. The lack of representation demonstrates healthcare disparities.

## 1. Introduction

The advent of tyrosine kinase inhibitors (TKIs) in the early 2000s revolutionized the treatment for chronic myeloid leukemia (CML), enabling substantial improvement in outcomes, leading to a near-normal life expectancy [1]. Over the course of their development in the last twenty years, TKIs have remained a mainstay of clinical trials and eventually standard treatment. However, despite their success, clinical trials evaluating TKIs have not been accessible to participants representative of the global CML patient population. Achieving equitable patient representation in clinical trials is of great interest in promoting health equity, as trial enrollment may allow patients to access treatment to which they otherwise would not have exposure to and healthcare providers with early experience with these treatments. Equitable representation is also paramount for enabling generalizability of trial outcomes, which should adequately demonstrate therapeutic effects in diverse populations. Since the passage of the National Institute of Health (NIH) Revitalization Act in 1993 for encouraging the inclusion of women and minorities in clinical research [2], several studies have demonstrated persisting demographic disparities in trial enrollment, preventing results from being representative of the patient population [3]. For example, in trials specifically evaluating therapies for leukemia, major disparities exist, such that non-Hispanic White patients are more likely to be enrolled in such trials than patients of other racial–ethnic identities [4,5]. Furthermore, the efficacy of targeted therapies such as TKIs may be influenced by the complex interactions of genetic differences associated with patients’ racial and ethnic ancestries and other factors (dietary, environmental, etc.) that may impact the pharmacodynamics and potentially the safety and efficacy of TKIs [6]. Pharmacodynamics refers to drug actions in a molecular, biochemical, and physiological manor [7]. Specifically, for imatinib, a greater frequency of serious adverse events was observed in Asian patients compared to non-Asian patients, attributed to higher imatinib plasma levels [8]. These observations strengthen the call to improve clinical trial representation of global patient populations.

Multinational participation in clinical trials may be limited by various socioeconomic factors that contribute to unequal research infrastructure and capacity across countries. The socio-demographic index (SDI) is a metric that quantifies income per capita, education, and female fertility rate for any given country or area in the Global Burden of Disease Study (GBDS) [9]. Since health and disease variables are not direct components of the SDI, the index is useful for considering socioeconomic determinants of health [10]. By placing countries’ clinical trial participation in the context of both their socioeconomic factors and their CML-affected patient populations, we stand to gain a better understanding of the relationship between the need for and availability of experimental TKIs.

Our objective in this study is to evaluate the extent to which phase 2 and phase 3 trials have representation from countries with different SDI and to evaluate TKI availability in those countries. We aim to assess the global trends of CML burden and the geographical distribution of pivotal phase 2 and phase 3 clinical trials for TKIs between 1999 and 2021. In doing so, we examine the relationship between low-, low-middle-, middle-, high-middle-, and high-SDI countries and available, approved TKIs. Through this study, we hope to find areas of need in both clinical trial representation and TKI availability.

## 2. Materials and Methods

### 2.1. Data Collection

Age-standardized CML incidence rates and disability-adjusted life years (DALYs) from 1999 to 2019 were obtained from the Institute of Health Metrics and Evaluation (IHME) database (University of Washington, Seattle, WA, USA). DALYs reflects a one-year loss of full health, with respect to the fact that mortality only is associated with death and notes the morbidity of certain conditions [11]. These data were specified by country and the World Bank regions. World Bank categorization was used as it had the most individual regions with seven of these, whereas the World Health Organization only had six regions. The regions include Europe and Central Asia (ECA), North America (NA), South Asia (SAsia), Middle East and North Africa (MENA), East Asia and Pacific (EAP), Sub-Saharan Africa (SSA), and Latin America and Caribbean (LAC). To assess trends over the 20-year period (1999–2019), we used Joinpoint regression which divides the data in time segments to identify years with statistically significant changes in trends. Joinpoint regression has been identified as an accurate method to estimate changes over time and provide the best-fit trend line across years of data, particularly for large population data [12,13]. The analysis was used to quantify results as annual percentage change (APC) between points and average annual percentage change for the DALYs and incidence rates, including 95% confidence intervals (95% CI). The Joinpoint Regression Program Version 5.0.2 (US National Cancer Institute, Rockville, MD, USA) was used for this analysis.

Global TKI approvals were collected from the drug’s brand pharmaceutical company as of September 2022: Novartis for imatinib, nilotinib, and asciminib; Bristol Myers Squibb for dasatinib; Pfizer for bosutinib; and Takeda for ponatinib. These were further quantified to match the number of approvals available for individual countries. The TKI approvals were presented by individual countries in their respective SDI categories of low, low-middle, middle, high-middle, and high, using data from IHME.

### 2.2. Clinical Trial Data

Clinical trial data were collected through a search on ClinicalTrials.gov (CT.gov) using the key words “Chronic Myeloid Leukemia”, “phase 2 trials”, and “phase 3 trials”. This generated 702 trials, 212 of which had results available on CT.gov. TKIs were used to treat CML in 52 of the 212 trials. Single-country (9; primarily in China, Japan, US, and Turkey) trials and early study termination (13) were excluded. This resulted in 30 eligible trials conducted between 1999 and 2019.

The city, state, country, and zip code of the investigational sites that participated in the 30 trials were also abstracted from CT.gov.

### 2.3. Statistical Analysis

The chi-square test for proportions and Fisher’s exact test (where appropriate) with 95% CI were used to assess the difference in proportion of DALYs in relation to the proportion of locations that participated in the trials, from each region. A *p*-value of 0.05 was considered statistically significant, and the SAS v9.4 (SAS Institute Inc., Cary, NC, USA) was used for statistical analyses. The difference in country-specific DALYs from 1999 to 2019 were also calculated as percent change. The frequency with which sites participated in the TKI trials were mapped and overlaid on the country-specific DALYs percent change using ArcGIS software (version 10.8.2). Geographical Information Software (GIS) methodologies were applied to investigate the spatial distribution and relationship between the location of the investigation sites of the trials and the countries in their respective SDI categories. The visual representation of the disease burden and inadequate access of important trials are communicated at a glance.

## 3. Results

### 3.1. Trends in Disability-Adjusted Life Years and Incidence Rates

Table 1 gives the Joinpoint analysis results for CML DALYs and incidence rates trends by region. The NA region had the highest significant decreases in DALYs at APCs of −8.72% (95% CI, −9.37, −8.35) from 1999 to 2004 and −5.42% (95% CI, −7.26, −3.71) from 2004 to 2007. ECA had significant declines between 1999 and 2013 with an APC of −4.32% (−4.50, −4.17), while LAC had significant APC decreases of −4.79% (−5.24, 3.84) from 2003 to 2006. NA (−3.72% (−3.8, −3.65)) and ECA (−3.34% (−3.46, −3.24)) also had the highest significant AAPC decreases over the entire period (1999–2019), while SAsia (−0.9% (−0.99, −0.8)) and SSA (−0.85% (−0.92, −0.77)) had the smallest decline.

**Figure 1:**
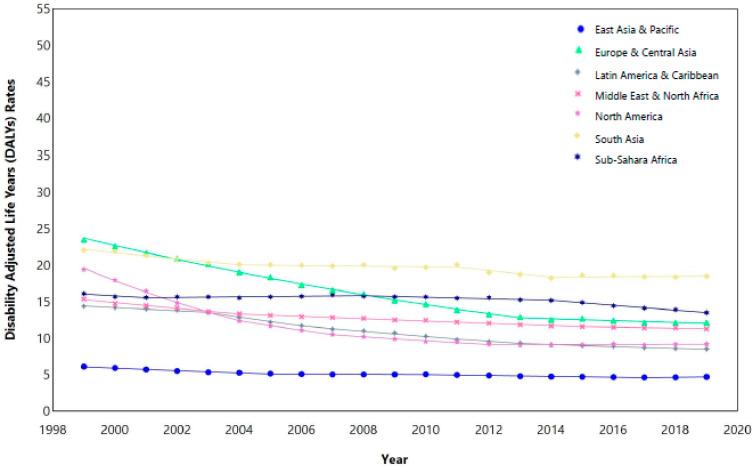

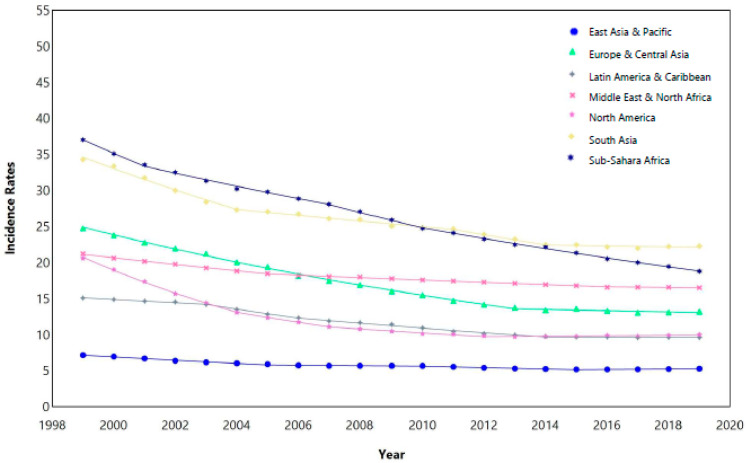
Trends in disability-adjusted life years (DALYs) and incidence rates of chronic myeloid leukemia by World Bank region, 1999–2019.

Similarly for incidence rates, NA had the highest decrease in rates at an APC of −8.75% (95% CI, −9.67, −8.31) from 1999 to 2004, then −5.47% (95% CI, −7.41, −3.51) from 2004 to 2007. Prominent significant declines were also noted for SSA at APCs of −5.08% (95% CI, −5.7, −4.11) from 1999 to 2001, SAsia at −4.59% (95% CI, −5.23, 4.16) from 1999 to 2004 and ECA at −4.23% (95% CI, 4.38, 4.1) from 1999 to 2013. NA (−3.6% (95% CI, −3.69, −3.51)), SSA (−3.3% (95% CI, −3.37, −3.25)), and ECA (−3.16% (95% CI, −3.26, −3.08)) had the highest significant AAPC decreases over the entire period (1999–2019), while MENA (−1.23% (95% CI, −1.26, −1.2)) had the smallest decline. Figure 1 shows the observed (points) for the DALYs and incidence rates with an overall declining trend over the 20-year period for all regions.

### 3.2. Tyrosine Kinase Inhibitor Trials Sites, Regions, and SDI Status

The 30 trials included in our analysis had investigational sites across 755 locations. Shown in Table 2 are the investigational sites for the respective World Bank regions and their corresponding incidence rates and DALYs for 2019. ECA had 344 of the trial locations (representing 45.6% of all trial locations), NA had 201 (26.6%), EAP had 121 (16%), LAC had 46 (6.1%), MENA had 19 (2.5%), SAsia had 13 (1.7%), and SSA had 11 (1.5%). The reported incidence rates per 100,000, from highest to lowest, were ECA at 3.01 (95% CI, 2.6–3.6), NA at 1.7 (95% CI, 1.5–2), SAsia at 0.62 (95% CI, 0.51–0.74), MENA at 0.57 (95% CI, 0.4–0.67), EAP at 0.45 (95% CI, 0.4–0.53), SSA at 0.44 (95% CI, 0.32–0.57), and LAC at 0.35 (95% CI, 0.3–0.4). DALYs per 100,000, from highest to lowest, were SAsia at 22.3 (95% CI, 18.5–27.0), SSA at 18.8 (95% CI, 13.5–25.7), MENA at 16.5 (95% CI, 11.3–20.2), ECA at 13.2 (95% CI, 12.1–15.2), NA at 10 (95% CI, 9.2–11.4), LAC at 9.6 (95% CI, 8.6–10.9), and EAP at 5.3 (95% CI, 4.7–6.3).

Table 2 also shows the chi-square test results for DALYs and trial sites. There were significant differences between the proportion of sites participating in relevant clinical trials and the DALYs for SAsia: 1.7% (95% CI, 0.8, 2.7) vs. 39.6 (95% CI, 10, 69.3) and SSA: 1.5% (95% CI, 0.6, 2.3) vs. 20∙1 (95% CI, 0, 44.3). A significant difference in the opposite direction (i.e., high proportion of sites participating in clinical trials and low DALYs) was seen in ECA: 45.6% (95% CI, 42, 49.1) vs. 11∙7 (95% CI, 0, 31.1).

At least one TKI was approved in 131 countries. Individual drug approvals included imatinib in 116 countries, dasatinib in 64, nilotinib in 124, bosutinib in 46, ponatinib in 60, and asciminib in 6. Table 3 shows the countries with drug approvals with respect to that country’s SDI status and DALYs. Thirty-one percent of the 131 countries with at least one TKI approved were high-SDI countries, 23% high-middle, 24% middle, 16% low-middle, and 7% were low-SDI countries. Furthermore, there were 178 individual drug approvals for the 6 drugs amongst the 40 high-SDI countries with TKI approvals for an average of 4.45 TKIs approved per high-SDI country. High-middle-SDI countries (30) had 108 approvals for the individual TKIs (average 3.6), middle-SDI countries (31) had 76 (2.45), low-middle-SDI countries (21) had 39 (1.85), and low-SDI countries (9) had 14 (1.55).

The 30 trials in this analysis included 29/40 (73%) of the high-SDI countries with drug approvals, 16/30 (53%) of the high-middle countries, 12/31 (39%) of the middle SDI countries, and only 1/21 (5%) of the low-middle-SDI countries, India. There were no low-SDI countries included in the 30 trials. Compared to other quintiles, DALYs rates were lowest in the high-SDI countries (1.1 × 10^5^) despite having the highest incidence rates (2.92 per 100,000). The highest DALYs were in the low SDI 2.6 × 10^5^ and low-middle SDI 3.1 × 10^5^ quintiles despite having the lowest incidence rates: 0.56 and 0.48 per 100,000, respectively.

### 3.3. Spatial Distribution of Tyrosine Kinase Inhibitor Trial Sites and DALYs

**Figure 2:**
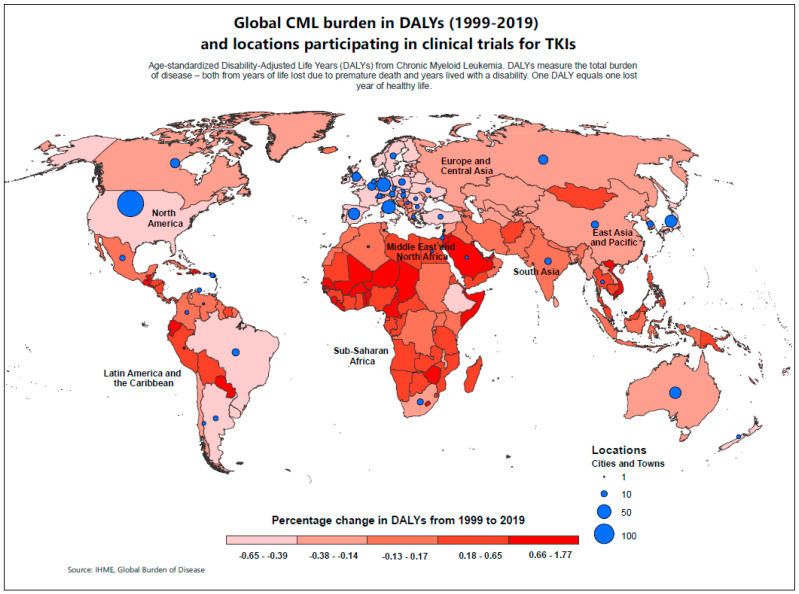
Spatial distribution of tyrosine kinase inhibitor clinical trial sites (1999–2021) and change in disability-adjusted life years (1999–2019).

Figure 2 shows the spatial distribution of sites that participated in the trials for TKIs and percent change (1999–2019) in DALYs for each country. Most countries in the African continent, parts of South Asia, including the islands and Mongolia, and many countries in LAC show higher percent increases over the 20-year period represented by the darker gradients. The United States, Brazil, Argentina, and some countries in Europe show the greatest decline in DALYs represented by the lighter gradients.

## 4. Discussion

Significant global misrepresentation is evident in clinical trial participation for TKIs, contributing to unequal availability of potentially life-saving investigational and eventual standard therapies. These trials are conducted predominantly in high-SDI countries, with most of the investigational sites being in NA and ECA. Though CML is diagnosed more frequently in these regions, as indicated by the higher incidence rates, the CML disease course is more severe and disabling in the regions with less clinical trial participation, indicated by higher DALYs in SAsia, SSA, and EAP. Over the past 20 years, SA has experienced both high DALYs and incidence rates (third in incidence rate after NA and ECA) without any significant decline in DALYs. With trends showing significantly higher decreases in incidence rates and DALYs in NA and other high-income countries or regions, consistent with prior studies [14,15]. Additionally, within the SSA region, participation was observed only from South Africa (middle SDI) in the 30 pivotal clinical trials leading to TKI approval. This shows a serious misrepresentation in involvement of African countries in these important trials. Similar distributions of CML burden in low-SDI regions were reported in results from the GBDS 2017 [16]. Overall, while global CML DALYs has decreased, the decline is primarily in high-SDI regions, while incidence and mortality has increased in low-SDI regions [15], amplifying the need to address underrepresentation in these clinical trials. It is possible that many patients go undiagnosed in low-SDI and perhaps other countries in the lower spectrum, accounting for some of the differences in incidence rates compared to NA and ECA. If this is the case, the disparities presented in our analysis would be further amplified.

In the context of clinical trials for cancer, similar trends in overrepresentation of the Caucasian/White population have been reported in other settings [17,18,19]. These regions have been previously evaluated in studies investigating causes of lower clinical trial participation in resource-limited areas, which identified barriers such as lack of financial capacity, research expertise and environment, and cumbersome ethical and regulatory systems [20,21,22]. Such systemic barriers may not only hamper research efforts but also may limit the extrapolation of available data to populations that have not been represented. Logistically, it may be challenging to establish investigational sites in resource poor regions given the extensive requirements for clinical trials. It is important, however, to understand and find ways to overcome these logistics and create pathways for clinical trials to be available. Specific trials designed for these patients and these regions (e.g., phase 4), and that are greatly simplified and designed with the intent to facilitate enrollment and follow up should be considered. Many of the same regions we identified as having underrepresentation for CML trials were also underrepresented during the rapid development and mobilization of COVID-19 clinical trials [23].

The findings from these critical trials should lead to the global availability of TKIs, but as we demonstrate, unfortunately, the approval of these drugs has a similar biased distribution to the trials. In our study, low-SDI countries had only 14 approvals ranging from 1 to 2 for the nine countries. This low number of TKI treatment options leaves few avenues in the event of treatment failure [24]. This is particularly pungent considering the challenges many of the underrepresented countries may face with establishing robust bone marrow transplantation programs and registries [25,26], coupled with the impracticality of long-distance travel for treatment due to individual-level financial barriers. The high cost of TKIs, once approved, and the uncertain quality of generic drugs used to fill the void, add to the disadvantages faced by patients in these underserved areas. This issue is not limited to clinical trials for CML. A recent publication on myeloma also showed significant global disparities based on income level and geography [27]. Furthermore, TKIs have revolutionized the treatment of CML, to the point where individuals can live long lives with this disease. Recent reports have demonstrated that a multitude of factors are critical for ensuring long lives in these patients, including individual characteristics, compliance, comorbidities, and centers familiar with these TKIs [28], as well as the ability to use next-generation TKIs in the event of multi-TKI failure or mutations (e.g., T315I) where only specific TKIs are expected to have possible efficacy.

### Limitations

Our analysis has limitations that should be acknowledged. It should be noted that the World Bank regions classification, although chosen because it breaks down regions more appropriately than the other subsets available on IHME, may have different healthcare conditions in different areas. For example, NA includes USA, Canada, and Mexico. Mexico has a different healthcare situation than the other two countries. Furthermore, although regions in the world may have similar economic characteristics, there are also areas where the SDI can be discordant and include differences in health care systems and policies. Pharmaceutical companies only have approvals for their brand-named drugs. Generic versions of many of these agents are available throughout the world. However, many of these generics may be of questionable quality, and safety and efficacy data from most of them, particularly in the countries with the greatest need, is largely unavailable. Generics are also limited to some TKIs, with the newest options not available even in generic formulations. For example, asciminib is only approved in a handful of countries but could be a useful medication to those who have failed multiple medications. Single-country trials were excluded. Some of these countries, particularly China, are heavily involved in the 30 trials to receive multiple drug approvals and may have agents available only in a few countries (e.g., radotinib and olverembatinib). There are also valuable programs that have made TKIs available to many countries in need. The Glivec International Patient Assistance Program (GIPAP), a collaboration between Novartis Pharma AG and the Max Foundation, has made imatinib available to more than 60,000 patients in more than 90 countries, mostly in the middle-low and low-SDI spectrum [29]. The 89% 5-year survival for patients enrolled in this program compares favorably to what has been reported from high-SDI countries and from clinical trials [30].

## 5. Conclusions

There is a lack of availability of TKIs at the global level. This directly affects patients with CML residing in underserved regions. In addition, the lack of equitable distribution of trials across countries is an issue that needs to be addressed. Increased representation of the global population, wider availability of drugs, and diagnostic abilities are fundamental to the advancement of CML research. Furthermore, systematic CML surveillance and reporting that is more representative of the global spectrum of patients is needed to more confidently ascertain whether the natural history of CML has been changed, for all. 

## Figures and Tables

**Table 1:** Trends in disability-adjusted life years and incidence of chronic myeloid leukemia by World Bank region, 1999–2019.

Region	Year	APC (95% CI)	Year	AAPC (95% CI)	Year	APC (95% CI)	Year	AAPC (95% CI)
Disability-Adjusted Life Years					Incidence			
East Asia and Pacific	1999–2005	−2.93 * (−3.56, −2.52)	1999–2019	−1.29 * (−1.42, −1.21)	1999–2005	−3.4 * (−3.8, −3.09)	1999–2019	−1.51 * (−1.59, −1.43)
	2005–2010	−0.33 (−1.44, 0.7)			2005–2010	−0.59 (0.99, 0.23)		
	2010–2017	−1.23 (−2.28, 0.92)			2010–2015	−1.77 * (2.58, 1.38)		
	2017–2019	1.04 (−0.59, 2.02)			2015–2019	0.58 * (0.03, 1.47)		
Europe and Central Asia	1999–2013	−4.32 * (−4.50, −4.17)	1999–2019	−3.34 * (−3.46, −3.24)	1999–2013	−4.23 * (−4.38, −4.1)	1999–2019	−3.16 * (−3.26, −3.08)
	2013–2019	−1.02 * (−1.55, −0.2)			2013–2019	−0.63 (−1.11, 0.04)		
Latin America and Caribbean	1999–2003	−1.54 * (−1.96, −0.82)	1999–2019	−2.6 * (−2.69, −2.51)	1999–2003	−1.55 * (−1.98, −0.88)	1999–2019	−2.24 * (−2.31, −2.16)
	2003–2006	−4.79 * (−5.24, −3.84)			2003–2006	−4.66 * (−5.09, 3.83)		
	2006–2013	−3.27 * (−3.61, −2.37)			2006–2014	−2.96 * (−3.15, −2.64)		
	2013–2019	−1.42 * (−1.77, −0.68)			2014–2019	−0.14 (−0.53, 0.37)		
Middle East and North Africa	1999–2004	−2.68 * (−3.24, −2.36)	1999–2019	−1.49 * (−1.56, −1.43)	1999–2005	−2.26 * (−2.33, −2.17)	1999–2019	−1.23 * (−1.26, −1.2)
	2004–2015	−1.28 * (−1.64, −1.16)			2005–2016	−0.93 * (−0.99, −0.9)		
	2015–2019	−0.6 (−1.04, 0.2)			2016–2019	−0.22 (−0.5, 0.21)		
North America	1999–2004	−8.72 * (−9.37, −8.35)	1999–2019	−3.72 * (−3.8, −3.65)	1999–2004	−8.75 * (−9.67, −8.31)	1999–2019	−3.6 * (−3.69, −3.51)
	2004–2007	−5.42 * (−7.26, −3.71)			2004–2007	−5.47 * (−7.41, −3.51)		
	2007–2012	−2.77 * (−3.37, −2.36)			2007–2012	−2.56 * (−3.27, −1.04)		
	2012–2019	0.07 (−0.2, 0.46)			2012–2019	0.33 * (0.02, 0.84)		
South Asia	1999–2004	−1.99 * (−2.86, −1.48)	1999–2019	−0.9 * (−0.99, −0.8)	1999–2004	−4.59 * (−5.23, −4.16)	1999–2019	−2.21 * (−2.3, −2.12)
	2004–2011	−0.22 (−0.5, 0.68)			2004–2011	−1.51 * (−1.73, −0.86)		
	2011–2014	−2.36 * (−2.92, −1.36)			2011–2014	−3.01 * (−3.51, −2.15)		
	2014–2019	0.15 (−0.28, 1.11)			2014–2019	−0.29 (−0.69, 0.45)		
Sub-Sahara Africa	1999–2001	−1.55 * (−2.2, −0.41)	1999–2019	−0.85 * (−0.92, −0.77)	1999–2001	−5.08 * (−5.7, −4.11)	1999–2019	−3.32 * (−3.37, −3.25)
	2001–2008	0.25 * (0.07, 0.97)			2001–2007	−2.84 * (−3.01, −2.29)		
	2008–2014	−0.68 * (−1.04, −0.37)			2007–2010	−3.99 * (−4.29, −3.43)		
	2014–2019	−2.32 * (−2.75, −2)			2010–2019	−3.03 * (−3.13, −2.79)		

Abbreviations: APC—annual percentage change, AAPC—average annual percentage change, * APC or AAPC in DALYs is significantly different from zero at *p* < 0.05.

**Table 2:** Regional chronic myeloid leukemia burden (IRs and DALYs) and difference in proportions for DALYs and investigational sites that participated in the TKI clinical trials.

Region	* IRs (95% CI)	* DALYs (95% CI)	Sites f, % (95% CI)	** DALYs (%)	χ^2^	*p*-Value
ECA	3.0 (2.6, 3.6)	13.2 (12.1,15.2)	344, 45.6 (42, 49.1)	11.7 (0, 31.1)	4.8	<0001 *
NA	1.7 (1.5, 2.0)	10 (9.2,11.4)	201, 26.6 (23.5, 29.8)	3.5 (0, 14.6)	2.8	0.0914
SAsia	0.62 (0.51, 0.74)	22.3 (18.5, 27)	13, 1.7 (0.8, 2.7)	39.6 (10, 69.3)	67.8	<0001 *
MENA	0.56 (0.40, 0.67)	16.5 (11.3, 20.2)	19, 2.5 (1.4, 3.7)	7.2 (0, 23)	0.9	0.3418
EAP	0.45 (0.40, 0.53)	5.3 (4.7, 6.3)	121, 16 (13.4, 18.6)	11.9 (0, 31.6)	0.1	0.7199
SSA	0.44 (0.32, 0.57)	18.8 (13.5, 25.7)	11, 1.5 (0.6, 2.3)	20.1 (0, 44.3)	21.2	<0001 *
LAC	0.35 (0.30, 0.40)	9.6 (8.6, 10.9)	46, 6.1 (4.4, 7.8)	6 (0, 20)	0.0	0.9888

Abbreviations: ECA—Europe and Central Asia; NA—North America; SAsia—South Asia; MENA—Middle East and North Africa; EAP—East Asia and Pacific; SSA—Sub-Saharan Africa; LAC—Latin America and Caribbean; DALY—disability-adjusted life years; IR—incidence rate, * Percent IRs and DALYs are for 2019 only. ** Percent DALYs calculated using regional total out of global total, *p*-value = 0.05.

**Table 3:** Tyrosine kinase inhibitor approvals (1999–2021) for chronic myeloid leukemia in relation to socio-demographic index, disability-adjusted life years, and incidence rates.

Low SDI	DALY	IR	Middle SDI	DALY	IR	High-Middle SDI	DALY	IR	High SDI	DALY	IR
(14 Approvals)	2.6 × 10^5^	0.56	(76 Approvals)	2.3 × 10^5^	0.32	(108 Approvals)	1.4 × 10^5^	0.96	(178 Approvals)	1.1 × 10^5^	2.92
Ethiopia (2)	8.4 × 10^4^	1.85	Albania (2)	1.4 × 10^2^	0.29	* Argentina (5)	4.3 × 10^3^	0.39	Aruba (2)	NR	NR
Ivory Coast (2)	3.5 × 10^3^	0.33	* Algeria (2)	6.9 × 10^3^	0.51	Bahrain (5)	3.6 × 10^3^	0.95	* Australia (5)	2.6 × 10^3^	1.88
Mali (2)	2.1 × 10^3^	0.22	Armenia (2)	2.5 × 10^2^	0.32	Bosnia-Herzegovina (1)	5.1 × 10^2^	0.80	* Austria (5)	1.3 × 10^3^	4.88
Pakistan (2)	5.5 × 10^4^	0.60	Azerbaijan (2)	6.6 × 10^2^	0.19	* Bulgaria (5)	1.0 × 10^3^	0.76	* Belgium (5)	1.3 × 10^3^	3.69
Senegal (2)	1.7 × 10^3^	0.29	Belarus (3)	3.6 × 10^3^	2.62	Chile (5)	1.9 × 10^3^	0.62	Brunei (2)	2.4 × 10^2^	1.63
Tanzania (1)	1.5 × 10^4^	0.56	Botswana (2)	1.3 × 10^2^	0.14	Croatia (5)	5.6 × 10^2^	2.17	* Canada (6)	3.7 × 10^3^	3.41
Togo (1)	9.6 × 10^2^	0.30	* Brazil (4)	1.7 × 10^4^	0.28	Georgia (2)	4.7 × 10^2^	0.42	Curacao (2)	NR	NR
Uganda (1)	6.7 × 10^3^	0.34	* China (2)	4.1 × 10^4^	0.26	* Greece (5)	2.1 × 10^3^	5.53	Cyprus (5)	6.0 × 10^1^	1.28
Yemen (1)	7.5 × 10^3^	0.60	* Colombia (5)	5.4 × 10^3^	0.44	* Hungary (5)	1.3 × 10^3^	1.17	* Czech Republic (5)	1.1 × 10^3^	1.48
			Costa Rica (2)	7.1 × 10^2^	0.71	* Israel (5)	7.1 × 10^2^	1.32	* Denmark (5)	7.1 × 10^2^	2.74
**Low-Middle SDI**	**DALY**	**IR**	Cuba (1)	3.0 × 10^3^	1.35	* Italy (4)	9.2 × 10^3^	6.74	* Estonia (5)	2.8 × 10^2^	2.21
(39 Approvals)	3.1 × 10^5^	0.48	Ecuador (3)	2.0 × 10^3^	0.36	Jordan (4)	2.7 × 10^2^	0.07	* Finland (5)	4.5 × 10^2^	1.41
Bangladesh (2)	2.7 × 10^4^	0.47	* Egypt (3)	1.6 × 10^4^	0.46	Kazakhstan (2)	2.2 × 10^3^	0.41	* France (5)	9.2 × 10^3^	5.04
Cameroon (2)	2.6 × 10^3^	0.21	Gabon (2)	2.4 × 10^3^	0.38	* Lebanon (5)	1.6 × 10^3^	2.11	* Germany (5)	1.6 × 10^4^	11.29
Dominican Republic (2)	7.2 × 10^2^	0.15	Indonesia (3)	1.9 × 10^4^	0.22	Macedonia (1)	1.7 × 10^2^	0.40	* Hong Kong (4)	NR	NR
El Salvador (2)	3.5 × 10^2^	0.19	Iraq (2)	5.2 × 10^3^	0.36	* Malaysia (3)	5.3 × 10^3^	0.54	Iceland (3)	2.3 × 10^1^	2.80
Ghana (2)	3.2 × 10^3^	0.27	Jamaica (2)	4.1 × 10^3^	0.47	Mauritius (2)	1.1 × 10^2^	0.32	* Ireland (5)	3.6 × 10^2^	3.40
Guatemala (2)	7.5 × 10^2^	0.13	Kosovo (1)	NR	NR	Moldova (2)	1.8 × 10^2^	0.18	* Japan (6)	1.1 × 10^4^	2.59
Honduras (2)	1.4 × 10^3^	0.46	* México (5)	1.1 × 10^4^	0.29	Montenegro (2)	1.2 × 10^2^	1.47	* South Korea (4)	1.3 × 10^3^	1.65
* India (2)	3.1 × 10^5^	0.64	Namibia (2)	5.9 × 10^1^	0.06	* Oman (4)	3.6 × 10^2^	0.32	Kuwait (3)	3.6 × 10^2^	0.25
Kenya (2)	4.0 × 10^3^	0.18	* Panama (2)	4.8 × 10^2^	0.39	* Poland (5)	5.4 × 10^3^	1.11	* Latvia (4)	3.4 × 10^2^	1.28
Kyrgyzstan (1)	3.6 × 10^2^	0.16	Paraguay (2)	6.6 × 10^2^	0.31	* Portugal (5)	1.3 × 10^3^	2.81	Liechtenstein (2)	NR	NR
Maldives (2)	3.1 × 10^1^	0.25	* Peru (4)	2.3 × 10^3^	0.22	* Romania (5)	1.4 × 10^3^	0.41	* Lithuania (5)	4.8 × 10^2^	0.95
Mongolia (1)	2.7× 10^2^	0.22	* Philippines (3)	1.0 × 10^4^	0.25	* Serbia (2)	9.4 × 10^3^	0.63	Luxembourg (5)	4.4 × 10^1^	2.49
Morocco (3)	2.9 × 10^3^	0.27	* South Africa (3)	1.0 × 10^3^	0.05	* Spain (5)	4.1 × 10^3^	3.68	Malta (5)	5.3 × 10^1^	2.85
Myanmar (1)	4.8 × 10^3^	0.25	Syria (1)	4.7 × 10^3^	1.12	Sri Lanka (2)	1.4 × 10^3^	0.23	* New Zealand (3)	4.2 × 10^2^	1.47
Nicaragua (2)	6.1 × 10^2^	0.31	* Thailand (4)	9.5 × 10^3^	0.49	Trinidad and Tobago (2)	1.8 × 10^2^	0.42	* Netherlands (5)	1.3 × 10^3^	3.41
Nigeria (2)	2.0 × 10^4^	0.22	* Tunisia (1)	1.1 × 10^3^	0.41	* Turkey (4)	7.3 × 10^3^	0.43	* Norway (3)	2.3 × 10^2^	1.02
Palestine (1)	5.5 × 10^2^	0.33	Turkmenistan (2)	4.0 × 10^2^	0.21	* Ukraine (3)	1.0 × 10^4^	0.98	Qatar (4)	3.6 × 10^2^	0.63
Sudan (2)	7.6 × 10^3^	0.46	Uzbekistan (2)	3.1 × 10^3^	0.26	Uruguay (3)	5.8 × 10^2^	0.76	* Russia (5)	2.3 × 10^4^	0.85
Tajikistan (1)	4.8 × 10^2^	0.13	Vietnam (2)	9.2 × 10^3^	0.32				* Saudi Arabia (5)	7.4 × 10^3^	0.72
Venezuela (3)	3.6 × 10^3^	0.44							* Singapore (4)	3.3 × 10^2^	1.18
Zimbabwe (2)	7.8 × 10^2^	0.12							* Slovak Republic (5)	4.2 × 10^2^	0.65
									Slovenia (5)	3.5 × 10^2^	3.86
									* Sweden (5)	7.5 × 10^2^	3.00
									* Switzerland (6)	9.4 × 10^2^	4.53
									* Taiwan (4)	2.0 × 10^3^	1.03
									* UAE (5)	1.9 × 10^3^	0.56
									* United Kingdom (5)	5.9 × 10^3^	2.61
									* USA (6)	3.3 × 10^4^	1.51

Abbreviations: SDI—socio-demographic index; UAE—United Arab Emirates; USA—United States of America; DAL—disability-adjusted life years; IR—incidence rate; NR—not reported. * Country is represented in the 30 global trials included in this study.

## Data Availability

The data that support the findings of this study are available upon request from the corresponding author, Jorge Cortes (jorge.cortes@augusta.edu).
